# Editorial: Advancements in AI-driven multimodal interfaces for robot-aided rehabilitation

**DOI:** 10.3389/frobt.2025.1605418

**Published:** 2025-05-15

**Authors:** Christian Tamantini, Kevin Patrice Langlois, David Rodriguez Cianca, Loredana Zollo

**Affiliations:** ^1^ National Research Council (CNR), Rome, Italy; ^2^ Research Unit of Advanced Robotics and Human-Centred Technologies, Campus Bio-Medico University, Rome, Italy; ^3^ Robotics and MultiBody Mechanics Research Group, Vrije Universiteit Brussel, Brussels, Belgium; ^4^ IMEC, Leuven, Belgium; ^5^ Neuroscience-Inspired AI and Robotics Research Group, Cajal International Neuroscience Center, Spanish National Research Council (CSIC), Madrid, Spain

**Keywords:** artifiical intelligence, multimodal data fusion, robot-aided rehabilitation, exoskeleton, robot control

In recent years, there has been a rapid evolution in the field of robot-aided rehabilitation, with transformative innovations being developed with the aim of supporting the recovery of individuals with motor impairments Mohebbi (2020). A particularly promising area of research is the development of Artificial Intelligence (AI)-driven multimodal interfaces, which have the capability to collect and interpret heterogeneous information from the patient and the environment Ai et al. (2021). By integrating signals from vision, force, motion, and physiological sensors, these interfaces enable a more objective, fine-grained, and dynamic understanding of motor performance and engagement during therapy Zhao et al. (2023). When coupled with AI, such systems can drive adaptive and personalized interventions, aligning robot behavior with the patient’s residual abilities and rehabilitation goals Adans-Dester et al. (2020).

This Research Topic explores the growing potential of multimodal sensor fusion, AI algorithms, and personalized control strategies to build intelligent rehabilitation platforms. The Research Topic includes four contributions: three literature reviews, and one original research article, each offering a unique perspective on the integration of AI and multimodal monitoring in robot-aided rehabilitation. The articles demonstrate how innovations in sensor design, signal interpretation, and patient modeling can lead to more responsive and effective robot-mediated therapies, enhancing patient outcomes while promoting evidence-based practice and large-scale data-driven insights.

Dalla Gasperina et al. review establishes the basis for comprehending the potential of human-robot physical interaction in the design of effective and cooperative rehabilitation strategies. Their multidisciplinary framework reviews the literature according to the various high-level training modalities, low-level control strategies, and hardware implementation proposed in the literature, emphasizing the necessity for control strategies that respond to the patient’s volitional effort and motor state.

In a complementary manner, the work of Vanmechelen et al. presents a comprehensive review of methodologies that can be used to assess upper limb movement disorders using wearable sensors. The study analyses wearable sensor technologies in different pathologies, highlighting how standardized protocols and sensitive sensor characteristics can support objective assessment of movement disorders and treatment monitoring. The work emphasizes the importance of real, non-intrusive monitoring to achieve meaningful rehabilitation results.

Furthermore, the contribution of Coser et al. concentrates on the lower limb domain, providing a comprehensive overview of the AI-based methods that can be utilized in exoskeleton-assisted rehabilitation. By systematically analyzing the application of AI models in robot control, intention detection, and locomotion classification, the authors emphasize the role of AI in facilitating task- and user-specific adaptation, with potential applications extending from clinical centers to telerehabilitation and home-based care. It is noteworthy that this paper was recognized among the top 11 publications of 2024 by Frontiers in Robotics and AI, signifying its significance and impact within the research community Kyriakopoulos (2025).

Complementing these literature analyses, Arora et al. propose an approach grounded on the use of a socially assistive robot to deliver neurorehabilitation training. Their work introduces an empathetic virtual coaching system integrated with a robotic device, designed to enhance patient engagement by sensing affective states and adapting the robot-therapy interaction accordingly. This novel combination of affective computing and socially interactive agents reintroduces the human-like relational dimension into robotic neurorehabilitation, which is critical especially in out-of-clinic settings.

The studies included in this Research Topic provide a solid foundation for guiding future research and development of novel AI-driven multimodal interfaces for rehabilitation robotics. The comprehensive reviews of the literature offer valuable references for researchers and practitioners in the field. Rather than focusing solely on technological innovation, these contributions help delineate key directions to be pursued to design truly effective and impactful rehabilitation systems. Furthermore, the original contribution underscores the nascent strides towards the integration of such technologies, encompassing artificial intelligence and multimodal patient monitoring within the clinical rehabilitation milieu.

A recurrent theme in the papers collected in this Research Topic is the significance of acquiring and integrating diverse data modalities to facilitate a more comprehensive and detailed comprehension of the patient’s motor and cognitive condition. This multimodal approach is pivotal to fostering personalization and adaptability in robot-assisted therapy. In addition, all contributions underscore the necessity for rigorous future clinical validation of AI-based methods, emphasizing that technological robustness alone is insufficient. It is imperative to demonstrate real-world utility and therapeutic impact for the effective integration of these innovations in clinical practice. Furthermore, all contributions converge on the importance of placing the patient at the center of the rehabilitation process. The promotion of active engagement is not only a matter of user experience but also a key factor in enhancing neuroplasticity and improving long-term outcomes.

By consolidating these insights, this Research Topic delineates a conceptual and methodological roadmap for future work, thereby fostering the development of intelligent, human-centered, and clinically meaningful solutions in robot-aided rehabilitation.
